# Effects of Group Counseling Programs, Cognitive Behavioral Therapy, and Sports Intervention on Internet Addiction in East Asia: A Systematic Review and Meta-Analysis

**DOI:** 10.3390/ijerph14121470

**Published:** 2017-11-28

**Authors:** Jun Liu, Jing Nie, Yafeng Wang

**Affiliations:** Department of Sociology & Institute for Empirical Social Science Research, School of Humanities and Social Sciences, Xi’an Jiaotong University, Xi’an 710049, China; liujunsoc@xjtu.edu.cn (J.L.); wyf.90.25.wyf@stu.xjtu.edu.cn (Y.W.)

**Keywords:** group counseling programs, cognitive behavioral therapy, sports intervention, Internet addiction, meta-analysis

## Abstract

To evaluate the effects of group counseling programs, cognitive behavioral therapy (CBT), and sports intervention on Internet addiction (IA), a systematic search in ten databases was performed to identify eligible studies without language restrictions up to January 2017. A meta-analysis and trial sequential analysis (TSA) was performed, respectively. A total of 58 randomized controlled trials (RCTs), which included 2871 participants, were incorporated into our meta-analysis. The results showed that group counseling programs, CBT, and sports intervention could significantly reduce IA levels (group counseling program: standardized mean difference (SMD), −1.37; 95% confidence interval (CI), −1.89 to −0.85; CBT: SMD, −1.88; 95% CI, −2.53 to −1.23; sports intervention: SMD, −1.70; 95% CI, −2.14 to −1.26). For group counseling programs, this treatment was more effective in four dimensions of IA, including time management, interpersonal and health issues, tolerance, and compulsive Internet use. For CBT, this treatment yielded a positive change in depression, anxiousness, aggressiveness, somatization, social insecurity, phobic anxiety, paranoid ideation, and psychoticism. For sports intervention, the significant effects were also observed in all dimensions of the IA scale. Each of group counseling programs, cognitive behavioral therapy, and sports intervention had a significant effect on IA and psychopathological symptoms. Sports intervention could improve withdrawal symptoms especially.

## 1. Introduction

With the Internet rapidly developing, IA (Internet addiction) has become considerable in public health, education, and relevant fields. IA is defined as excessive and compulsive Internet use which causes distress and serious results in social and occupational problems [1]. Over the last 15 years, the number of Internet users has increased rapidly, IA has become a widespread and problematic phenomenon. Currently, it is estimated that IA affects 1.5–8.2% of the general population in the United States and Europe [2,3]. The prevalence of IA has increased rapidly in East Asia. IA rates of were 10.7% for teenagers in Korea and approximately 10% of adolescents reported a tendency towards, or current diagnosis of, IA in China [4,5].

IA has been increasingly recognized as a serious mental disorder, which can result in a cluster of cognitive and behavioral symptoms, including progressive loss of time management skills, interpersonal skills, health, compulsive Internet use, tolerance, and withdrawal symptoms. Moreover, it may cause psychological distress and psychological disturbances, like social anxiety, depression, anxiousness, and low self-esteem [6]. Recently, more and more studies have investigated the effects of psychological, pharmacological, and sports interventions on IA and psychopathological symptoms. Four meta-analyses assessed the effects of the prevention and intervention treatment for IA [7,8,9,10]. Winkler et al. found that psychological and pharmacological interventions were effective treatments for reducing symptoms of IA, time spent online, anxiety, and depression [8]. The review by Yeun et al. suggested that psychosocial intervention might be used to prevent Internet addiction in school-aged children [7]. Park et al. evaluated the effects of group counseling programs in Korea [9], and Oh et al. performed a meta-analysis in terms of the effects of a program of IA prevention and interventions in teenagers [10].

However, the included studies were limited and insufficient samples were not powerful enough to support the results in the previous meta-analysis; few studies focused on the prevention and treatment of IA on psychopathological symptoms, such as anxiety, depression, anxiousness, and low self-esteem.

Therefore, we conducted a meta-analysis with trial sequential analysis (TSA) to estimate the efficacy of different types of treatments on IA and psychopathological symptoms. We also evaluated whether there was a relapse after group counseling programs. Furthermore, stratified analyses were also performed to evaluate their effects in college students and secondary school students.

## 2. Materials and Methods

### 2.1. Literature Search

This systematic review and meta-analysis was conducted and reported according to the PRISMA Statement [11]. A computerized search of The Cochrane Library, PubMed, MEDLINE, EMBASE, Scopus, Web of Science, PsycINFO, Chinese BioMedical Literature, China National Knowledge Infrastructure, and WANFANG databases from inception to September 2016 was conducted to identify the relevant studies by two blinded authors (Jun Liu and Jing Nie) independently. The search strategy was based on a combination of the terms (“Internet addiction”, “Internet usage”, “problematic Internet”, “computer game addiction”, “online addiction”, “Internet overuse” or “Internet disorder”) and (“treat”, “treatment”, “therap”, “training”, “psychotherap”, “program”, “curricul”, or “workshop”). The searches were with no language restrictions. In addition, the reference lists of eligible studies and previous evidence summaries also were reviewed to identify additional literature. When data were inadequate for necessary analysis, the relevant supplements and appendixes would be checked for the eligible data.

### 2.2. Study Selection

The included studies in the meta-analysis had to meet the following inclusion criteria: (1) study design was limited to randomized controlled trials; (2) participants were diagnosed with Internet addiction; (3) no interventions or usual care were given to the control group; (4) the study objective was to evaluate the effect of treatment on Internet addiction; and (5) the study included psychological, physical, pharmacological, or comprehensive programs which implement several different types of treatments intended to decrease IA-related problems. Studies would be excluded if they did not fulfill the following criteria: (1) the study was a single-arm pre-post comparison study; (2) the full text of the study was unavailable; or (3) the study did not provide sufficient data or original data. Moreover, for multiple studies based on the same sample or overlapping data, the ones with the most subjects would be included. Based on these criteria, two independent authors (Jing Nie and Yafeng Wang) selected the studies, and any discrepancies were resolved by discussion to achieve consensus.

### 2.3. Outcome Measurement

Assessment tools of Internet addiction varied between studies and used various questionnaire scales which included Young Internet Addiction Scale (YIAS), Young Diagnostic Questionnaire for Internet Addiction (YDQ), Chinese Internet Addiction Scales Revision (CIAS-R), Chinese Internet Addiction Scale (CIAS), Korea-Internet Addiction Scale (KIAS), Adolescent Pathological Internet Use Scale (APIUS), Internet Overuse Self-Rating Scale (IOSR) and Internet Addiction Scale (IAS). Psychopathological symptoms were assessed by Symptom Check List-90 (SCL-90), Self-Rating Anxiety Scale (SAS), Self-Rating Depression Scale (SDS), Hamilton Depression Scale (HMDS), Self-rated Health Measurement Scale Version l.0 (SRHMS), and Self-Esteem Scale (SES).

### 2.4. Data Abstraction and Quality Assessment

Two independent reviewers (Jing Nie and Yafeng Wang) abstracted data using a standardized data extraction form. The following information was collected: author, publication year, cultural background, total sample size, the number of participants in different groups, hours of intervention, follow-up period, grade, diagnostic tools of Internet addiction assessment tools, outcome variables assessment tools, and mean and standard deviation of outcome variables.

Risk of bias was assessed by two reviewers (Jing Nie and Yafeng Wang) using Cochrane collaboration risk assessment tools for randomized controlled trials [12]. This tool consisted of the following seven domains: random sequence generation, allocation concealment, blinding of participants and researchers, blinding of outcome assessment, incomplete outcome data, selective reporting, and other bias. Individual domains were estimated as ‘low risk of bias’, ‘unclear’, or ‘high risk of bias’ by two independent reviewers.

### 2.5. Statistical Analysis

The difference change between the intervention group and comparison group was calculated as the effect size of intervention on Internet addiction. Due to studies reporting effect sizes by varying scales, the standardized mean difference (SMD) was calculated [13]. A Cochran’s Q statistic and the I^2^ test were performed to determine the homogeneity of this study. When significant homogeneity(*p* < 0.1 and I^2^ > 50%) was present, a random-effect model was performed for meta-analysis. Otherwise, a fixed-effect model would be chosen. To investigate potential sources of heterogeneity, we conducted stratified analyses. In addition, sensitivity analysis by sequentially removing one study at a time was performed to assess the stability of the pooled results. Potential publication bias was examined by the Begg’s funnel plot and Egger’s regression test, with *p* < 0.10 taken as an indication of publication bias.

A meta-analysis involves repeated significance testing on accumulating data which may increase the risk of type I errors which cause false positive or negative results. Based on the problem mentioned above, it is possible that TSA can be adopted to minimize the random errors and analyze whether the available sample was powered enough to support the results. In our study, the required information size (RIS) was calculated and TSA monitoring boundaries were built based on an overall 5% risk of a type I error, 20% of the type II error (a statistical test power of 80%). If the cumulative Z-curve has crossed the trial sequential monitoring boundaries or below the futility boundaries before the required information size is reached, robust evidence might have been confirmed and no further studies are necessary, whereas it is necessary to continue performing trials. If the Z curve of the cumulative meta-analysis crossed the trial sequential monitoring boundaries or below the futility boundaries before the required information size is reached, further randomization is not necessary, and there is sufficient information to support the conclusions. When none of the two boundaries and the RIS line are crossed by the Z-curve, evidence is relatively insufficient to draw a conclusion [14].

The main analyses were performed using STATA version 11.0 (Stata Corp LP, College Station, TX, USA). A *p* value of <0.05 was considered statistically significant. TSA were conducted using TSA software version 0.9 (beta) (Copenhagen Trial Unit, Copenhagen, Denmark).

## 3. Results

### 3.1. Identification and Characteristics of Eligible Studies

**Figure 1:**
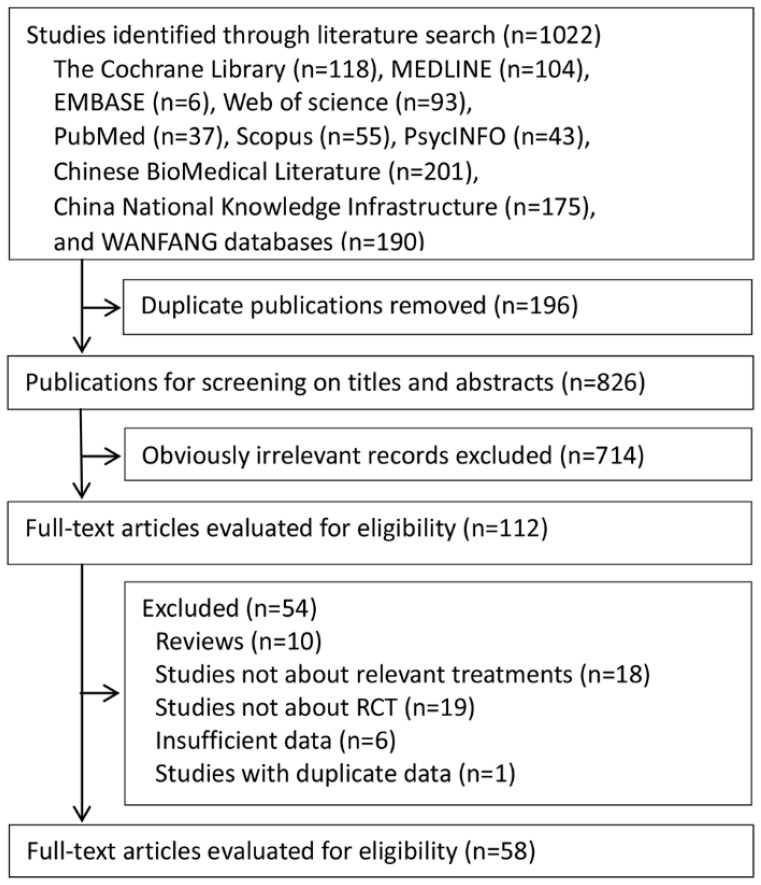
The flowchart of study inclusion and exclusion.

As a result of the first bibliographic search, a total of 1022 potentially eligible articles were retrieved. After deleting the duplicates, 826 papers were selected for screening titles and abstracts, with 112 determined to be potentially eligible (Figure 1). After reviewing the full articles and contacting the authors to obtain the necessary data, 58 articles were included in our meta-analysis (Table S1).

The characteristics of the included studies were summarized in Table 1. Of the total 2871 participants, the intervention group included 1408 patients, and the control group contained 1463 patients. All studies were randomized controlled trials. Among the included studies, 57 studies were performed in China and one was conducted in Korea. Thirty of these treatments were group counseling programs, 15 were CBT, and 13 were sports intervention. Among CBT, nine were individual therapies and six were group therapies. All studies were conducted in adolescents, except for six studies that contained a mixture of adolescents and adults. Total number of hours spent in treatment ranged from 4 to 80. Duration of intervention varied from four weeks to 48 weeks. The longest available follow-up period was six months. The summary results of the risk of bias are shown in Table S2.

### 3.2. Effects of Group Counseling Programs

**Figure 2:**
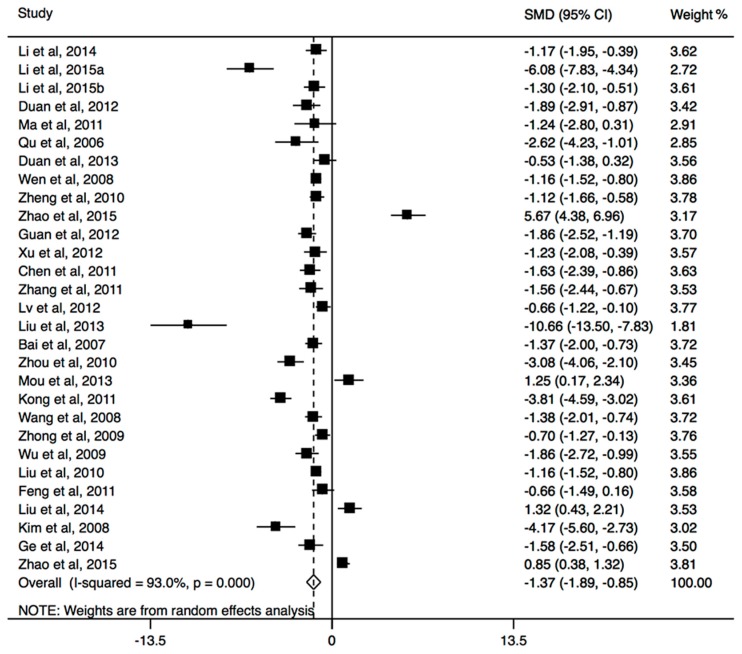
The forest plot of efficacy of group counseling programs on Internet addiction level.

All thirty included studies with group counseling programs measured IA or its subscales, and eleven involved psychopathological symptoms. When the relevant studies were pooled into the meta-analysis, the results showed that significant decrease in the score of IA is observable in group counseling programs (SMD, −1.37; 95% CI, −1.89 to −0.85; Figure 2), with significant heterogeneity (I^2^ = 93.0%, *p* < 0.001). The pooled results of four studies providing both immediate and follow-up assessment, did not find a relapse (SMD, −0.10; 95% CI, −0.40 to 0.20; Table 2). This treatment was more effective than control group in four dimensions of IA scale (time management: SMD, −0.42; 95% CI, −0.76 to −0.09; interpersonal and health issues: SMD, −0.93; 95% CI, −1.50 to −0.37; tolerance: SMD, −0.58; 95% CI, −1.04 to −0.11; compulsive Internet use: SMD, −0.65; 95% CI, −1.15 to −0.15; Table 2). For the dimension of withdrawal symptoms, no significant effect was observed among the treatment (SMD, −0.53; 95% CI, −1.38 to 0.32). In addition, the results of subgroup analysis showed that the significant effect of this program was observed in both college students and secondary school students, and both short-term and long-term programs could significantly reduce Internet addiction (Table 2). In psychopathological symptoms, the group counseling program yielded significant change in SCL-subscales obsessive-compulsive (SMD, −0.97; 95% CI, −1.76 to −0.17), depression (SMD, −1.61; 95% CI, −2.96 to −0.25), anxiousness (SMD, −1.58; 95% CI, −3.12 to −0.05), and aggressiveness (SMD, −2.29; 95% CI, −4.39 to −0.19), but no significant effects were found in the Global Severity Index (GSI) and other subscales including somatization, social insecurity, phobic anxiety, paranoid ideation, and psychoticism (All *p* > 0.05). The self-efficacy expectancy did not increase significantly after the treatment (SMD, 0.50; 95% CI, −0.62 to 1.62). The Begg’s funnel plot and Egger’s regression test did not find any evidence for the presence of publication bias (both *p* > 0.1).

### 3.3. Effects of CBT

**Figure 3:**
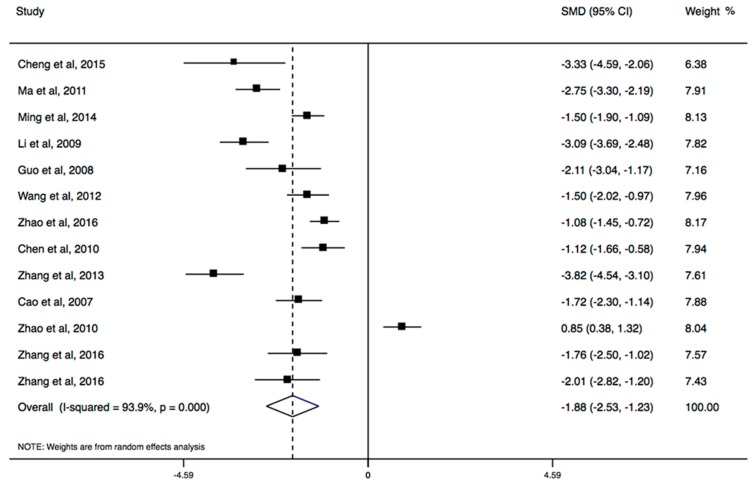
The forest plot of efficacy of cognitive behavioral therapy on Internet addiction level.

Thirteen of all included studies with CBT measured IA or its subscales, and six were involved in psychopathological symptoms. The results of the meta-analysis showed that CBT had a large effect for reducing IA (SMD, −1.88; 95% CI, −2.53 to −1.23; Figure 3), regardless of individual treatment or group treatment, but CBT did not have a significant effect in five dimensions of the IA scale (Table 3). The longer program could have a significant effect on IA (SMD, −1.80; 95% CI, −2.33 to −1.26). In psychopathological symptoms, CBT yielded a positive change in GSI, depression, anxiousness, aggressiveness, somatization, social insecurity, phobic anxiety, paranoid ideation, and psychoticism, but not obsessive-compulsive disorder (Table 3). There was no evidence for the presence of publication bias (*p* > 0.05).

### 3.4. Effects of Sports Intervention

**Figure 4:**
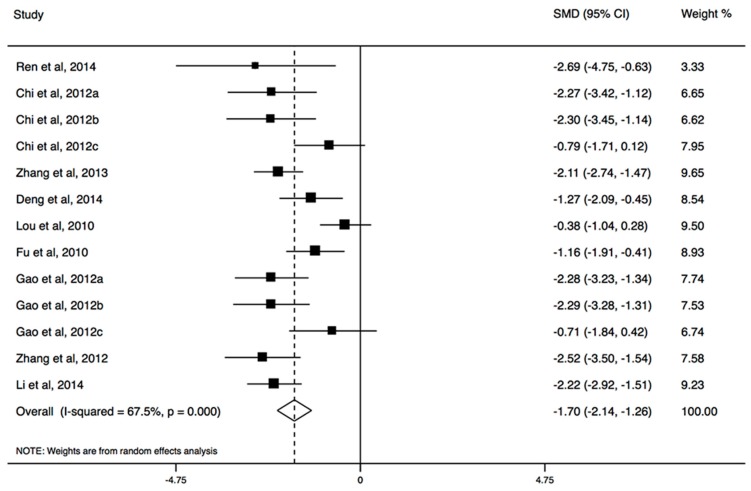
The forest plot of efficacy of sports intervention on Internet addiction level.

Nine of the included studies with group counseling programs measured IA or its subscales, and six involved in psychopathological symptoms. The results of the meta-analysis showed that sports had a large effect for reducing IA (SMD, −1.70; 95% CI, −2.14 to −1.26; Figure 4), especially for mild and moderate IA (Table 4). The significant effects were also observed in all dimensions of IA scale (Table 4). The effect size in college students for IA was larger than that in secondary school students. Both short-term and long-term programs could significantly reduce IA (Table 4). In psychopathological symptoms, sports could improve GSI, depression, anxiousness, aggressiveness, somatization, social insecurity, phobic anxiety, paranoid ideation, and psychoticism, but not obsessive-compulsive disorder (Table 4). There was no strong evidence of systematic publication bias (*p* > 0.05).

### 3.5. Trial Sequential Analysis

**Figure 5:**
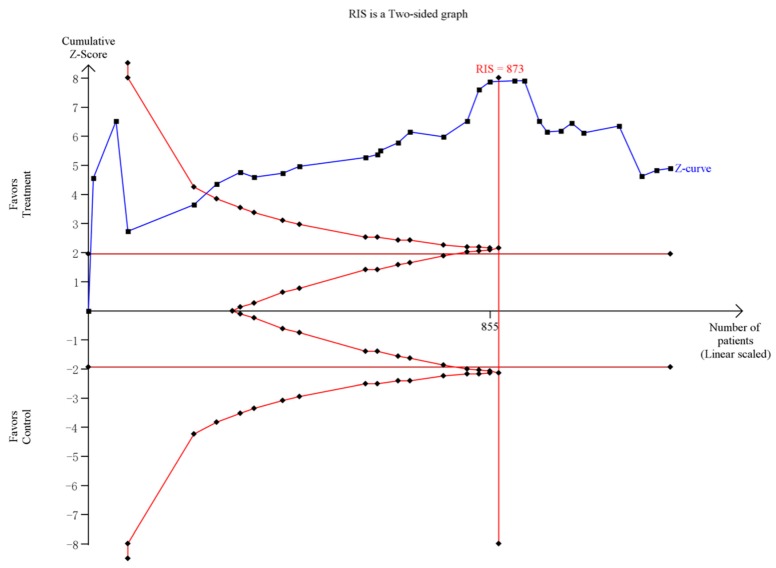
Trial sequential analysis of efficacy of group counseling program on Internet addiction level. The blue line represents the cumulative Z-score of the meta-analysis. The straight red represents the conventional *p* = 0.05 statistical boundaries. The inward sloping red lines represent the truncated trial sequential monitoring boundaries.

**Figure 6:**
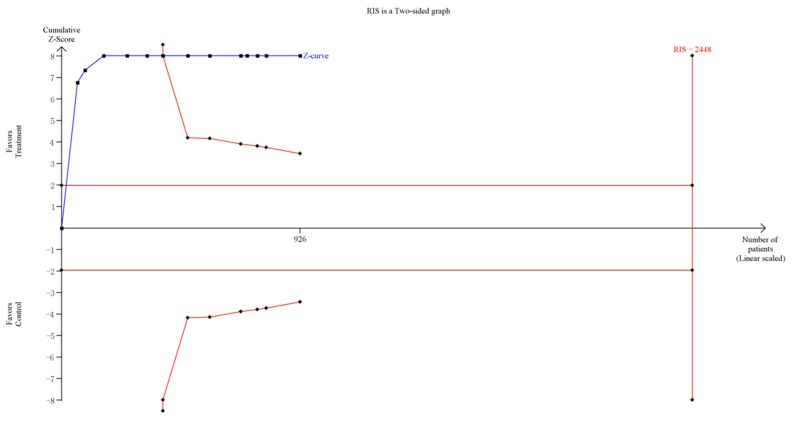
Trial sequential analysis of efficacy of cognitive behavioral therapy on Internet addiction level. The blue line represents the cumulative Z-score of the meta-analysis. The straight red line represents the conventional *p* = 0.05 statistical boundaries. The inward sloping red lines represent the truncated trial sequential monitoring boundaries.

**Figure 7:**
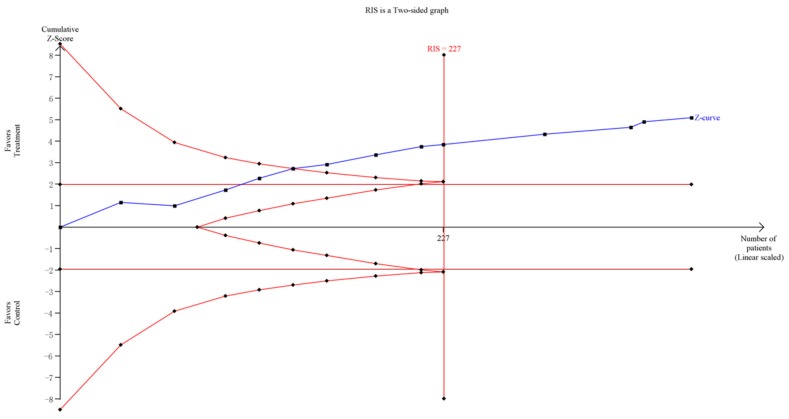
Trial sequential analysis of efficacy of sports intervention on Internet addiction level. The blue line represents the cumulative Z-score of the meta-analysis. The straight red line represents the conventional *p* = 0.05 statistical boundaries. The inward sloping red lines represent the truncated trial sequential monitoring boundaries.

For group counseling programs, an RIS of 873 subjects was calculated. The Z-curve crossed the trial sequential monitoring boundaries and it demonstrated that there was sufficient evidence to support the significant effect of group counseling programs on IA (Figure 5). For CBT, the result of the TSA showed the RIS is 2448 subjects. Although the accrued number of patients did not reach RIS, the cumulative Z-curve crossed the conventional significance test boundary and the effect was established previously. For psychological nursing intervention, TSA and RIS adjusted boundary values cannot be calculated due to limited information size (Figure 6). For sports intervention, the RIS is 227 subjects to demonstrate the issue. The Z-curve crossed the trial sequential monitoring boundaries, suggesting the significant effect of sports on IA had been confirmed (Figure 7). Overall, according to TSA, the power of the study was sufficient for efficiency of group counseling programs, CBT, and sports intervention on IA, and further relevant trials were unnecessary.

## 4. Discussion

Our results demonstrated the efficacy of group counseling program, cognitive behavioral therapy, and sports intervention for patients suffering from Internet addiction (IA) compared to those with usual care or no special intervention. Different types of interventions might reduce symptoms of IA through improving different dimensions of interpersonal and health issues, time management, tolerance, and compulsive Internet use. In addition, the different types of interventions could have different beneficial effects on psychopathological symptoms of somatization, social insecurity, obsessive-compulsive disorder, depression, anxiousness, aggressiveness, paranoid ideation, and psychoticism, indicating that an individual therapy might be administered to patients who were IA and had different psychopathological symptoms.

IA was associated with potential detrimental side-effects, and the patients suffering from IA also had mental health problems, such as loneliness, low self-esteem, anxiety, and depression [15,16,17]. Especially for students, they spent more time playing computer games or other IA behaviors, which would lead to a lack of sleep and higher levels of tiredness [18]. Started in the early 20th century, group counseling programs could make the group members know themselves and help themselves, increase social participation and interpersonal communication among the patients, improve adaptation, develop personality, and then eliminate the symptoms of IA [19]. Group counseling programs could gain patients confrontation and insight from experiencing similar cognitions and emotions, which was demonstrated the predominant modality for treating IA [20]. The meta-analysis showed that group counseling programs could improve interpersonal and health issues, time management, tolerance, and compulsive Internet use. In addition, deterioration of mental status could be ameliorated in obsessive-compulsive disorder, depression, anxiousness, and aggressiveness. The previous meta-analysis by Park et al. identified the beneficial effect of group counseling programs for teenagers with IA [9], which was in agreement with our results. Moreover, we compared the short-term and long-term efficacies of group counseling programs for IA and identified the robustness of their efficacy.

Cognitive behavioral therapy (CBT) emphasized the association of behaviors with thoughts and emotions, and impelled patients to pay more attention to these, and their thoughts and feelings, would make them identify addictive behavior triggers. CBT was identified to be effective in treating IA in many studies, which was in agreement with our study [21,22,23]. The results of the present meta-analysis showed that both individual and group CBT had beneficial effects on secondary school students and college students with IA, respectively. In addition, CBT could significantly improve depression, anxiousness, aggressiveness, somatization, social insecurity, phobic anxiety, paranoid ideation, and psychoticism.

The patients with IA spent large amounts of time on the Internet, which would reduce their attention, cause them brain fatigue, and degrade their physical function. The previous studies showed sports could improve blood and oxygen supply in the cerebrum, enhance the excitability of the cerebral cortex, and strengthen the balance and flexibility of the nervous system, which would improve the human body function and psychological adaptability [24,25]. Therefore, sports intervention had several potential benefits over IA treatment approaches. In our study, sports intervention improved depression, anxiousness, aggressiveness, somatization, social insecurity, phobic anxiety, paranoid ideation, and psychoticism. In addition, except for improving interpersonal and health issues, time management skill, tolerance, and compulsive Internet use, withdrawal symptoms were also improved after treatment. This beneficial effect on withdrawal symptoms were not found in group counseling programs and CBT.

Several potential limitations should also merit consideration in interpreting the findings of the present study. First, although the included studies were all randomized controlled trials, most of them had a high risk bias which might affect the robustness of our results; however, the total sample size is relatively large, and the results of the trial sequential analysis revealed that the power of study was sufficient and the robust evidence might have been confirmed, which adds to the strength of our analysis. Second, as reported in a previous review [8], IA intervention studies tended to lack conformity, with inconsistencies in the definition and diagnosis of IA, which confused the results; moreover, due to studies reporting effect sizes by varying scales, standardized mean difference (SMD) would be chosen to calculate the outcome variables; thus, we only evaluated whether the treatment is effective and whether the efficacy is larger, and did not quantify the magnitude of this effect. Therefore, a uniform diagnosis and assessment tool was necessary in the future study. Third, it is possible that populations with specific cultural backgrounds may affect the analysis of the relationship, but all included studies in the present meta-analysis were performed in China, except for one study conducted in Korea, and there were no studies available in the United States or Europe which indicated that IA is rapidly increasing in East Asia; therefore, there was a lack of conclusive evidence of cultural differences in IA and, therefore, further large research studies with randomization or blinding techniques are also necessary to estimate the efficacy of different types of treatments on IA and psychopathological symptoms in Europe and US. Fourth, genetic factors played a role on generalized IA, as well as on specific facets, such as self-regulation, online social interaction, or negative consequences. Our analysis was based primarily on data and information provided from the original literature and genetic ascendancies regarding IA were not discussed in our review. Therefore, further research should consider genetic factors and their influence on IA. Finally, potential publication bias was also a concern. Although apparent publication bias was not observed by statistical tests, it was still difficult to be solved completely because there was not a sufficient number of studies to detect it adequately.

## 5. Conclusions

The present meta-analysis identified that all of the group counseling programs, cognitive behavioral therapies, and sports interventions had a significant effect on Internet addiction (IA) and psychopathological symptoms. The efficacy of group counseling program remained robustness after immediate treatment and follow-up. Sports intervention could improve withdrawal symptoms, especially. Further carefully-designed studies are warranted to evaluate the efficacy of different treatments in different populations and to estimate the magnitude of genetic influences on IA.

## Figures and Tables

**Table 1:** Characteristics of the included studies.

Author	Country	Total N	tNt0/tNt1	cNt0/cNt1	Hours of Intervention Applied	Participants	Follow up	Type of Treatment (Group/Individual)	Diagnostic Tools of Internet Addiction	Outcome Variables (Assessment Tools)
Li et al., 2014 [Table S1:1]	China	30	15/15	15/15	12	College student	NR	Group counseling program (group)	CIAS-R	IA and its subscales (CIAS-R); psychopathological symptoms (SCL-90)
Li et al., 2015 [Table S1:2]	China	60	30/30	30/30	116	College student	2 months	Group counseling program (group)	CIAS	IA and its subscales (CIAS)
Duan et al., 2012 [Table S1:3]	China	22	11/11	11/11	52–65	College student	3 months	Group counseling program (group)	YIAS	IA and its subscales(YIAS)
Ren et al., 2014 [Table S1:4]	China	8	4/4	4/4	54–72	College student	NR	Sports (group)	YIAS	IA (YIAS); psychopathological symptoms (SCL-90)
Cheng et al.,2015 [Table S1:5]	China	23	12/12	11/11	24	College student	NR	CBT (individual)	YIAS	IA (YIAS)
Zhao et al., 2015 [Table S1:6]	China	77	36/32	45/45	NR	College student	1 month	Group counseling program (group)	YDQ	IA and its subscales (CIAS,YDQ); psychopathological symptoms (SCL-90); SE (SES)
Ma et al., 2011 [Table S1:7]	China	8	4/4	4/4	NR	College student	NR	Group counseling program (group)	YIAS	IA (YIAS)
Qu et al., 2006 [Table S1:8]	China	12	6/6	6/6	NR	College student	NR	Group counseling program (group)	YIAS	IA (YIAS)
Ma et al., 2011 [Table S1:9]	China	98	49/49	49/49	NR	College student	NR	CBT (individual)	YIAS	IA (YIAS)
Duan et al., 2013 [Table S1:10]	China	22	11/11	11/11	65	College student	NR	Group counseling program (group)	YIAS	IA and its subscales (YIAS)
Wen et al., 2008 [Table S1:11]	China	140	60/60	80/80	40	College student	3 months	Group counseling program (group)	YIAS	IA (YIAS)
Zheng et al., 2010 [Table S1:12]	China	61	30/30	31/31	6.6	College student	NR	Group counseling program (group)	YDQ	IA (YDQ); psychopathological symptoms (SCL-90)
Zhao et al., 2015 [Table S1:13]	China	48	24/24	24/24	16	College student	NR	Group counseling program (group)	CIAS-R	IA (CIAS-R)
Chi et al., 2012 [Table S1:14]	China	20	10/10	10/10	60	College student	NR	Sports (group)	YIAS	IA (YIAS); psychopathological symptoms (SCL-90)
Guan et al., 2012 [Table S1:15]	China	50	25/25	25/25	26	College student	3 months	Group counseling program (group)	CIAS	IA (CIAS)
Ming et al., 2014 [Table S1:16]	China	120	60/60	60/60	120–160	College student	NR	CBT (individual)	CIAS, YDQ	IA (YIAS); psychopathological symptoms (SCL-90)
Xu et al., 2012 [Table S1:17]	China	26	13/13	13/13	24	Secondary school student	1 week	Group counseling program (group)	IAS	IA (IAS-R); psychopathological symptoms (SDS, SAS)
Chen et al., 2011 [Table S1:18]	China	37	14/14	23/23	NR	College student	NR	Group counseling program (group)	YIAS, CIAS-R	IA and its subscales (CIAS-R); psychopathological symptoms (SCL-90)
Zhang et al., 2013 [Table S1:19]	China	60	30/30	30/30	64	College student	NR	Sports (group)	YIAS	IA(YIAS)
Zhang et al., 2011 [Table S1:20]	China	26	13/13	13/13	10.7	Secondary school student	NR	Group counseling program (group)	APIUS	IA and its subscales (APIUS); psychopathological symptoms (MHSMS)
Li et al., 2009 [Table S1:21]	China	92	48/44	48/48	NR	Adolescents	Post-treatment; 8 weeks	CBT (individual)	YDQ	IA (CIAS, YDQ); psychopathological symptoms (SCL-90)
Guo et al., 2007 [Table S1:22]	China	18	9/9	9/9	16	Adolescents	NR	Sports (group)	YIAS, IAS	Psychopathological symptoms (SCL-90)
Guo et al., 2008 [Table S1:23]	China	28	16/14	16/14	8–8.8	College student and secondary school student	NR	CBT (group)	CIAS, YDQ	IA (CIAS); psychopathological symptoms (SCL-90)
Guo et al., 2006 [Table S1:24]	China	18	9/9	9/9	16	Adolescents	NR	CBT (individual)	YIAS, IAS	Psychopathological symptoms (SCL-90)
Lv et al., 2012 [Table S1:25]	China	52	26/26	26/26	12	College student	6 weeks	Group counseling program (group)	CIAS	IA (CIAS)
Wang et al., 2012 [Table S1:26]	China	72	36/36	36/36	12	College student	NR	CBT (individual)	IAS	IA (YDQ)
Ming et al., 2014 [Table S1:27]	China	58	28/28	30/30	20–30	College student	NR	CBT (individual)	CIAS-R, YDQ	Psychopathological symptoms (SCL-90)
Zhao et al., 2010 [Table S1:28]	China	47	36/32	45/45	120–160	College student	1 month	CBT (individual)	YDQ	IA and its subscales (CIAS, YDQ)
Zheng et al.,2013 [Table S1:29]	China	41	20/20	21/21	21–28	College student	Post-treatment; 3 months	Group counseling program (group)	YIAS, CIAS	Psychopathological symptoms (SCL-90); SE (SES)
Deng et al., 2014 [Table S1:30]	China	48	24/24	24/24	25	College student	NR	Sports (group)	CIAS	IA (CIAS); psychopathological symptoms (SCL-90)
Zhang et al., 2009 [Table S1:31]	China	32	16/16	16/16	NR	Secondary school student	Immediate	Sports (group)	CIAS	IA and its subscales (CIAS); psychopathological symptoms (SCL-90)
Wu et al., 2013 [Table S1:32]	China	16	8/8	8/8	28–42	College student	Immediate	Sports (group)	YDQ	Psychopathological symptoms (SCL-90)
Lou et al., 2010 [Table S1:33]	China	36	18/18	18/18	54–72	Secondary school student	Immediate	Sports (group)	YDQ	IA(YDQ)
Fu et al., 2010 [Table S1:34]	China	32	16/16	16/16	>24	Secondary school student	NR	Sports (group)	YIAS	IA (YIAS); psychopathological symptoms (SCL-90)
Gao et al., 2012 [Table S1:35]	China	69	35/35	34/34	135	College student	NR	Sports (group)	YIAS	IA (YIAS); psychopathological symptoms (SCL-90)
Liu et al., 2013 [Table S1:36]	China	32	16/16	15/15	24	College student	1 week	Group counseling program (group)	YIAS	IA (YIAS)
Bai et al., 2007 [Table S1:37]	China	48	24/24	24/24	16	College student	Post-treatment; 6 weeks	Group counseling program (group)	CIAS-R	IA (CIAS-R)
Zhou et al., 2010 [Table S1:38]	China	18	9/9	9/9	16	College student	NR	Group counseling program (group)	CIAS	IA (CIAS)
Zhao et al., 2016 [Table S1:39]	China	130	65/65	65/65	96	Adolescents	NR	CBT(group)	CIAS-R	IA (CIAS)
Mou et al., 2013 [Table S1:40]	China	16	8/8	8/8	12	College student	NR	Group counseling program (group)	CIAS-R, YDQ	IA (CIAS-R)
Kong et al., 2011 [Table S1:41]	China	71	36/36	35/35	40	College student	NR	Group counseling program (group)	YIAS	IA (YIAS)
Wang et al., 2008 [Table S1:42]	China	48	24/24	24/24	24	College student	Post-treatment; 1 month; 3 months	Group counseling program (group)	CIAS, YDQ	IA (CIAS)
Zhong et al., 2009 [Table S1:43]	China	51	28/28	24/24	NR	Adolescents	NR	Group counseling program (group)	OCS	IA (OCS)
Wu et al., 2009 [Table S1:44]	China	30	15/15	15/15	NR	College student	NR	Group counseling program (group)	CIAS, APUIS	IA and its subscales (CIAS-R, APIUS); psychopathological symptoms (SCL-90); SE (SES)
Liu et al., 2010 [Table S1:45]	China	160	80/80	80/80	40	College student	3 month	Group counseling program (group)	YIAS	IA (YIAS)
Chen et al., 2010 [Table S1:46]	China	61	30/30	31/31	6.6	College student	NR	CBT (group)	YDQ	IA (YDQ); psychopathological symptoms (SCL-90)
Ge et al., 2014 [Table S1:47]	China	24	12/12	12/12	10	Children	Post-treatment; 1 month; 3 months	Group counseling program (group)	CIAS	IA and its subscales (CIAS)
Wang et al., 2009 [Table S1:48]	China	112	56/56	56/56	48	Adolescents	NR	Group counseling program (group)	YDQ	Psychopathological symptoms (SCL-90, SDS, SAS)
Zhang et al., 2013 [Table S1:49]	China	84	42/42	42/42	NR	College student	NR	CBT (individual)	CIAS	IA and its subscales (CIAS)
Feng et al., 2011 [Table S1:50]	China	24	12/12	12/12	24	College student	NR	Group counseling program (group)	IAS	IA (IAS) and its subscales; psychopathological symptoms (SRHMS)
Liao et al., 2008 [Table S1:51]	China	50	20/20	30/30	35	College student	Immediate	Sports (group)	CIAS	IA (CIAS); psychopathological symptoms (SCL-90)
Zhang et al., 2012 [Table S1:52]	China	42	22/22	20/20	30	College student	NR	Sports (group)	CIAS	IA and its subscales (CIAS)
Liu et al., 2014 [Table S1:53]	China	24	12/12	12/12	16	Secondary school student	1 week	Group counseling program (group)	IAS	IA (IAS)
Cao et al., 2007 [Table S1:54]	China	57	29/26	35/31	8–12	Secondary school student	NR	CBT (group)	YDQ	IA (CIAS, YDQ)
Li et al., 2014 [Table S1:55]	China	51	27/27	24/24	30	Secondary school student	NR	Sports (group)	YIAS	IA (YIAS)
Kim et al., 2008 [Table S1:56]	Korea	25	13/13	12/12	12.5	College student	NR	Group counseling program (group)	K-IAS	IA and its subscales (K-IAS); SE (SES)
Zhang et al., 2016 [Table S1:57]	China	40	23/23	17/17	16.47	NR	NR	CBT (group)	CIAS	IA (CIAS)
Zhang et al., 2016 [Table S1:58]	China	26	20/20	16/16	15	NR	NR	CBT (group)	CIAS	IA (CIAS)

APIUS: Adolescent Pathological Internet Use Scale; CBT: Cognitive Behavioral Therapy; CIAS: Chinese Internet Addiction Scale; CIAS-R: Chinese Internet Addiction Scales Revision; KIAS: Korea-Internet Addiction Scale; HMDS: Hamilton Depression Scale; IAS: Internet Addiction Scale; IOSR: Internet Overuse Self-Rating Scale; NA: Not available; NR: Not reported; YIAS: Young Internet Addiction Scale; YDQ: Young Diagnostic Questionnaire for Internet Addiction; SAS: Self-Rating Depression Scale; SCL-90: Symptom Check List-90; Self-Rating Anxiety Scale: SDS; SES: Self-Esteem Scale; SRHMS: Self-rated Health Measurement Scale Version l.0.

**Table 2:** Effect sizes for all outcome variables for group counseling program.

Outcome	*N*	SMD	Lower	Upper	Q	I^2^	*p*
IA	30	−1.37	−1.89	−0.85	412.08	93.00%	*p* < 0.001
Relapse	4	−0.10	−0.40	0.20	0.2	0.00%	0.98
College student	24	−1.52	−2.11	−0.92	365.29	93.70%	*p* < 0.001
Secondary school student	2	−1.39	−2.00	−0.78	0.27	0.00%	0.61
Long-term	8	−1.88	−2.65	−1.11	74.82	90.60%	*p* < 0.001
Short-term	15	−1.21	−2.09	−0.33	239.94	94.20%	*p* < 0.001
Time management	10	−0.42	−0.76	−0.09	19.36	53.50%	0.02
Interpersonal and health issues	9	−0.93	−1.50	−0.37	40.67	80.30%	*p* < 0.001
Tolerance	11	−0.58	−1.04	−0.11	43.04	76.80%	*p* < 0.001
Withdrawal symptoms	6	−0.53	−1.38	0.32	41.51	88.00%	*p* < 0.001
Compulsive Internet use	10	−0.65	−1.15	−0.15	41.15	78.10%	*p* < 0.001
GSI	6	−0.47	−0.98	0.04	16.11	69.00%	0.01
Somatization	4	−1.71	−4.13	0.71	138.2	97.80%	*p* < 0.001
Obsessive-compulsive	5	−0.97	−1.76	−0.17	30.47	86.90%	*p* < 0.001
Social insecurity	4	−1.59	−3.34	0.16	78.56	96.20%	*p* < 0.001
Depression	6	−1.61	−2.96	−0.25	99.27	96.00%	*p* < 0.001
Anxiousness	6	−1.58	−3.11	−0.05	124.68	96.80%	*p* < 0.001
Aggressiveness	5	−2.29	−4.39	−0.19	144.61	97.20%	*p* < 0.001
Phobic anxiety	4	−0.83	−2.41	0.75	74.62	96.00%	*p* < 0.001
Paranoid ideation	5	−0.98	−2.18	0.21	64.28	93.80%	*p* < 0.001
Psychoticism	4	−0.73	−1.97	0.50	47.69	93.70%	*p* < 0.001
SES	4	0.50	−0.62	1.62	33.44	91.00%	*p* < 0.001

GSI: Global Severity Index across nine subscales of Symptom Check List-90; IA: Internet addiction; SES: Self-Esteem Scale; SMD: standardized mean difference.

**Table 3:** Effect sizes for all outcome variables for cognitive behavioral therapy.

Outcome	*N*	SMD	Lower	Upper	Q	I^2^	*p*
IA	13	−1.88	−2.53	−1.23	198.25	93.90%	*p* < 0.001
Group	7	−1.53	−1.89	−1.16	185.64	96.80%	*p* < 0.001
Individual	6	−1.88	−2.53	−1.23	10.3	51.40%	0.07
College student	7	−1.84	−2.95	−0.73	162.57	96.30%	*p* < 0.001
Secondary school student	1	−2.08	−3.45	−0.70	0	NA	*p* < 0.001
Long-term	7	−1.80	−2.33	−1.26	40.98	85.40%	*p* < 0.001
Short-term	3	−1.68	−4.78	1.42	120.95	98.30%	*p* < 0.001
Time management	2	−0.74	−2.49	1.02	27.26	96.30%	*p* < 0.001
Interpersonal and health issues	2	−0.94	−3.42	1.54	48.76	97.90%	*p* < 0.001
Tolerance	2	−0.67	−3.09	1.75	48.72	97.90%	*p* < 0.001
Withdrawal symptoms	1	0.91	0.43	1.38	0	NA	*p* < 0.001
Compulsive Internet use	2	−1.14	−4.25	1.98	68.36	98.50%	*p* < 0.001
GSI	4	−2.24	−3.60	−0.88	76.40	96.10%	*p* < 0.001
Somatization	4	−1.22	−1.51	−0.93	1.79	0.00%	0.62
Obsessive-compulsive	4	−0.57	−1.17	0.04	11.48	73.90%	0.01
Social insecurity	4	−1.46	−2.70	−0.21	38.75	92.30%	*p* < 0.001
Depression	4	−1.93	−3.33	−0.52	42.27	92.90%	*p* < 0.001
Anxiousness	4	−1.48	−2.75	−0.21	40.11	92.50%	*p* < 0.001
Aggressiveness	4	−1.01	−1.63	−0.38	11.38	73.60%	0.01
Phobic anxiety	4	−1.27	−2.01	−0.54	14.66	79.50%	*p* < 0.001
Paranoid ideation	4	−1.04	−1.64	−0.43	10.57	71.60%	0.01
Psychoticism	4	−1.69	−2.82	−0.56	30.12	90.00%	*p* < 0.001

GSI: Global Severity Index across nine subscales of Symptom Check List−90; IA: Internet addiction; NA: not available; SES: Self-Esteem Scale; SMD: standardized mean difference.

**Table 4:** Effect sizes for all outcome variables for sports intervention.

Outcome	*N*	SMD	Lower	Upper	Q	I^2^	*p*
IA	8	−1.70	−2.14	−1.26	28.48	67.50%	*p* < 0.001
Mild IA	3	−2.6	−3.44	−1.75	2.91	31.40%	0.23
Moderate IA	3	−2.35	−3.00	−1.69	0.08	0.00%	0.96
Serious IA	2	−0.76	−1.47	−0.05	0.01	0.00%	0.91
College student	6	−1.89	−2.30	−1.48	10.64	34.20%	0.16
Secondary school student	2	−1.70	−2.73	−0.66	4.04	75.20%	0.04
Long-term	4	−1.92	−2.37	−1.47	6.79	26.30%	0.24
Short-term	3	−1.98	−2.70	−1.27	4.50	55.50%	0.11
Time management	3	−0.93	−1.32	−0.53	0.43	0.00%	0.81
Interpersonal and health issues	3	−0.34	−1.30	0.63	11.88	83.20%	*p* < 0.001
Tolerance	3	−0.95	−1.52	−0.37	4.06	50.80%	0.13
Withdrawal symptoms	3	−1.07	−1.48	−0.67	1.57	0.00%	0.46
Compulsive Internet use	3	−2.58	−4.54	−0.61	28.21	92.90%	*p* < 0.001
GSI	3	−1.01	−1.55	−0.47	0.52	0.00%	0.77
Somatization	5	−0.79	−1.15	−0.44	1.6	0.00%	0.81
Obsessive-compulsive	5	−0.51	−1.05	0.03	8.69	53.90%	0.07
Social insecurity	5	−0.64	−1.07	−0.21	5.67	29.50%	0.23
Depression	5	−0.85	−1.20	−0.49	1.19	0.00%	0.88
Anxiousness	5	−0.90	−1.26	−0.55	2.82	0.00%	0.59
Aggressiveness	5	−0.62	−0.97	−0.27	3.45	0.00%	0.49
Phobic anxiety	5	−0.52	−0.97	−0.08	6.23	35.80%	0.18
Paranoid ideation	5	−0.60	−0.95	−0.26	2.38	0.00%	0.67
Psychoticism	5	−0.53	−0.97	−0.08	6.15	34.90%	0.19

GSI: Global Severity Index across nine subscales of Symptom Check List-90; IA: Internet addiction; SES: Self-Esteem Scale; SMD: standardized mean difference.

## Supplementary Materials

The following are available online at www.mdpi.com/1660-4601/14/12/1470/s1; Table S1: Studies included in the meta-analysis, Table S2: Risk of bias assessment for randomized trials.

## References

[1] Pontes H.M., Macur M., Griffiths M.D. (2016). Internet Gaming Disorder among Slovenian Primary Schoolchildren: Findings From a Nationally Representative Sample of Adolescents. J. Behav. Addict..

[2] Mohammadbeigi A., Valizadeh F., Mirshojaee S.R., Ahmadli R., Mokhtari M., Ghaderi E., Ahmadi A., Rezaei H., Ansari H. (2016). Self-rated Health and Internet Addiction in Iranian Medical Sciences Students, Prevalence, Risk Factors and Complications. Int. J. Biomed. Sci..

[3] Weinstein A., Lejoyeux M. (2010). Internet addiction or excessive internet use. Am. J. Drug. Alcohol Abus..

[4] Wu X.S., Zhang Z.H., Zhao F., Wang W.J., Li Y.F., Bi L., Qian Z.Z., Lu S.S., Feng F., Hu C.Y. (2016). Prevalence of Internet addiction and its association with social support and other related factors among adolescents in China. J. Adolesc..

[5] Lee C.S., McKenzie K. (2015). Socioeconomic and Geographic Inequalities of Internet Addiction in Korean Adolescents. Psychiatry Investig..

[6] Seyrek S., Cop E., Sinir H., Ugurlu M., Şenel S. (2017). Factors associated with Internet addiction: Cross-sectional study of Turkish adolescents. Pediatr. Int. Off. J. Jpn. Pediatr. Soc..

[7] Yeun Y.R., Han S.J. (2016). Effects of Psychosocial Interventions for School-aged Children’s Internet Addiction, Self-control and Self-esteem: Meta-Analysis. Healthc. Inform. Res..

[8] Winkler A., Dörsing B., Rief W., Shen Y., Glombiewski J.A. (2013). Treatment of internet addiction meta-analysis. Clin. Psychol. Rev..

[9] Park S.M. (2009). A meta-analysis on the effects of adolescent internet addiction group counseling program in Korea. Korean J. Couns. Psychother..

[10] Oh I.S., Kim C. (2009). Meta-analysis on the effects of the prevention and intervention programs for internet addiction. J. Korean Assoc. Inf. Educ..

[11] Moher D., Liberati A., Tetzlaff J., Altman D.G., PRISMA Group (2009). Preferred reporting items for systematic reviews and meta-analyses, the PRISMA statement. BMJ.

[12] West C.P., Dyrbye L.N., Erwin P.J., Shanafelt T.D. (2016). Interventions to prevent and reduce physician burnout systematic review and meta-analysis. Lancet.

[13] Lee J., Lee Y., Gong S., Bae J., Choi M. (2016). A meta-analysis of the effects of non-traditional teaching methods on the critical thinking abilities of nursing students. BMC Med. Educ..

[14] Wetterslev J., Thorlund K., Brok J., Gluud C. (2008). Trial sequential analysis may establish when firm evidence is reached in cumulative meta-analysis. J. Clin. Epidemiol..

[15] Koyuncu T., Unsal A., Arslantas D. (2014). Assessment of internet addiction and loneliness in secondary and high school students. J. Pak. Med. Assoc..

[16] Li W., Zhang W., Xiao L., Nie J. (2016). The association of Internet addiction symptoms with impulsiveness, loneliness, novelty seeking and behavioral inhibition system among adults with attention-deficit/hyperactivity disorder (ADHD). Psychiatry Res..

[17] Bozoglan B., Demirer V., Sahin I. (2013). Loneliness, self-esteem, and life satisfaction as predictors of Internet addiction cross-sectional study among Turkish university students. Scand. J. Psychol..

[18] Chen Y.L., Gau S.S. (2016). Sleep problems and internet addiction among children and adolescents longitudinal study. J. Sleep Res..

[19] Kim J.U. (2015). The effect of a R/T group counseling program on the Internet addiction level and self-esteem of Internet addiction university students. Forum Soc. Econ..

[20] Park S.Y., Kim S.M., Roh S., Soh M.A., Lee S.H., Kim H., Lee Y.S., Han D.H. (2016). The effects of a virtual reality treatment program for online gaming addiction. Comput. Methods Programs Biomed..

[21] Ong S.H., Tan Y.R. (2014). Internet addiction in young people. Ann. Acad. Med. Singap..

[22] Du Y.S., Jiang W., Vance A. (2010). Longer term effect of randomized, controlled group cognitive behavioural therapy for Internet addiction in adolescent students in Shanghai. Aust. N. Z. J. Psychiatry.

[23] Santos V., Nardi A.E., King A.L. (2015). Treatment of internet addiction in patient with panic disorder and obsessive compulsive disorder case report. CNS Neurol. Disord. Drug Targets.

[24] Rupp T., Jubeau M., Millet G.Y., Wuyam B., Levy P., Verges S., Perrey S. (2013). Muscle, prefrontal, and motor cortex oxygenation profiles during prolonged fatiguing exercise. Adv. Exp. Med. Biol..

[25] Morris J.P., Pelphrey K.A., McCarthy G. (2005). Regional brain activation evoked when approaching a virtual human on a virtual walk. J. Cogn. Neurosci..

