# Supplementary material for: Effects of Group Counseling Programs, Cognitive Behavioral Therapy, and Sports Intervention on Internet Addiction in East Asia: A Systematic Review and Meta-Analysis
Source: Int J Environ Res Public Health. 2017 Nov 28;14(12):1470. doi: 10.3390/ijerph14121470 (PMC5750889; doi:10.3390/ijerph14121470)
Supplement: Supplementary file 1 [file ijerph-14-01470-s001.pdf]

Table S1: Studies included in the meta-analysis.

Number	Study	Title
1	Li et al, 2014	The effects of Adlerian group counseling on college students with Internet addiction
2	Li et al, 2015	Effects of group counseling on Internet addiction among vocational medical students
3	Duan et al, 2012	Psychological intervention on Internet dependence in college students and its effect: based on the control test in human police academy
4	Ren et al, 2014	The study of sport intervention experiment on college students with Internet addiction
5	Cheng et al, 2015	The intervention effect of hypnosis combined with cognitive behavioral therapy to cyber deviant behavior
6	Zhao et al, 2015	Internet addiction college students: social-psychological factors and group psychological intervention
7	Ma et al, 2011	Study of psychological intervention of college students' Internet addiction disorder
8	Qu et al, 2006	Study of the psychological mechanism college students' Internet addiction and psychological intervention
9	Ma et al, 2011	Case-control study of cognitive behavior in the tendency of Internet addiction and loneliness among the undergraduates
10	Duan et al, 2013	The investigations, intervention of Internet addiction in college students -taking human police academy as an example
11	Wen et al, 2008	The research of group counseling on Internet-dependent college students
12	Zheng et al, 2010	Compared study of group psychotherapy in College Students with Internet addiction
13	Zhao et al, 2015	Empirical research of group counseling to college students Internet addiction
14	Chi et al, 2012	Internet addiction college students sports experimental study of intervention in city of Changchun
15	Guan et al, 2012	Effect of group therapy on higher vocational school students with Internet dependence
16	Ming et al, 2014	Evaluation study of cognitive-behavioral therapy to Internet addiction intervention effects of medical students
17	Xu et al, 2012	Research on the interference of Internet addiction on the basis of self-controlled group guidance
18	Chen et al, 2011	Evaluation of intervention effects of periodical group counseling to college students Internet addiction
19	Zhang et al, 2013	A study on the sports intervention of undergraduates'Internet addiction in Kunming city
20	Zhang et al, 2011	Internet addiction investigate and group psychological study of country junior high school
21	Li et al, 2009	Cognitive-behavior therapy on adolescent with Internet addiction disorder
22	Guo et al, 2007	A study on psychotherapy and exercise prevention for Internet addiction and disorder among adolescents
23	Guo et al, 2008	Impact evaluation of group counseling on Internet addiction adolescents

24	Guo et al, 2006	A study on psychotherapy and exercise prevention on Internet addiction disorder of adolescents
25	Lv et al, 2012	The effects of group counseling on Internet-dependent college students
26	Wang et al, 2012	Evaluation of intervention effect of Internet addiction by cognitive behavioral therapy for college students
27	Ming et al, 2014	The effect of cognitive behavioral therapy on the intervention of Internet addiction behavior of medical students
28	Zhao et al, 2010	Effects of cognitive behavioral training on college students Internet addiction disorder
29	Zheng et al, 2013	The efficacy of neuro linguistic programming group counseling on university freshmen with tendency of Internet addiction
30	Deng et al, 2014	Study on the intervention of physical exercise on the Internet addiction of higher vocational college students
31	Zhang et al, 2009	Study on the intervention of physical exercise on the Internet addiction of adolescents
32	Wu et al, 2013	Empirical study about using sports means to intervene in Internet addiction of college students
33	Lou et al, 2010	An empirical study on the intervention effect of physical exercise prescription on teenage Internet addiction
34	Fu et al, 2010	Experimental study on sports intervention of adolescent Internet addiction
35	Gao et al, 2012	Influence of sport interference on university students'network addiction
36	Liu et al, 2013	Effects of Internet addiction group counseling on Internet using and study management in college students
37	Bai et al, 2007	The effects of group counseling on Internet-dependent college students
38	Zhou et al, 2010	Study on intervention effects of group counseling on Internet-dependent college students
39	Zhao et al, 2016	Control study of curative effect on Internet addiction disorder elimination by cognitive-behavioral therapy in groups
40	Mou et al, 2013	Intervention effects of group sand play therapy on undergraduate with Internet dependence
41	Kong et al, 2011	Effect of group guidance on Internet addiction disorder among university students
42	Wang et al, 2008	Research on the effect of group counseling intervention on adolescents' network dependence
43	Zhong et al, 2009	Effect of group psychological intervention in adolescents on Internet addiction
44	Wu et al, 2009	The study of group training for weakening higher vocational students Internet addiction tendency
45	Liu et al, 2010	Effects of centralized and closed group counseling on Internet-dependent undergraduates
46	Chen et al, 2010	Group psychotherapy among college students with Internet addiction in Wenzhou
47	Ge et al, 2014	Effects of psychodrama group counseling on internet addiction and social avoidance in urban left-behind children
48	Wang et al, 2009	Effect of psychological drama therapy on negative emotion of patients with Internet addiction

49	Zhang et al, 2013	The study of interventions cognitive therapy to medical students with Internet addiction disorder
50	Feng et al, 2011	The study of group counseling intervention on students’ network addiction by using core self-evaluation
51	Liao et al, 2008	Intervention of aerobic exercise for Internet addiction college students
52	Zhang et al, 2012	Effect of exercise prescription on college students' Internet dependence symptom
53	Liu et al, 2014	Moral education evaluation based on educational dialogue-the trend of moral education evaluation reform under the dialogue paradigm of moral education evaluation
54	Cao et al, 2007	Control study of group psychotherapy on middle school students with Internet overuse
55	Li et al, 2014	Current situation and exercise prescription intervention of Internet addiction among middle school students
56	Kim et al, 2008	The Effect of a R/T Group Counseling Program on The Internet Addiction Level and Self-Esteem of Internet Addiction University Students
57	Zhang et al, 2016	Effects of craving behavioral intervention on neural substrates of cue-induced craving in Internet gaming disorder
58	Zhang et al, 2016	Altered resting-state neural activity and changes following a craving behavioral intervention for Internet gaming disorder

Table S2: Risk of bias assessment for randomised trials.

Study	Random sequence generation	Allocation concealment	Blinding of participants and researchers	Blinding of outcome assessment	Incomplete outcome data	Selective reporting	Other bias
Li et al, 2014	Unclear	Unclear	Unclear	Low	Low	Low	Low
Li et al, 2015	Unclear	Unclear	Unclear	Low	Low	Low	Low
Duan et al, 2012	Unclear	Low	Unclear	Low	Low	Unclear	Low
Ren et al, 2014	Low	Unclear	Unclear	Low	Low	Low	Low
Cheng et al, 2015	Unclear	Unclear	Unclear	Low	Unclear	Low	Low
Zhao et al, 2015	Low	Low	Unclear	Low	Low	Low	Low
Ma et al, 2011	Low	Low	Unclear	Low	Low	Low	Low
Qu et al, 2006	Unclear	Low	Unclear	Low	Low	Low	Low
Ma et al, 2011	Low	Unclear	Unclear	Low	Low	Low	Low
Duan et al, 2013	Low	Unclear	Unclear	Low	Low	Low	Low
Wen et al, 2008	Low	Unclear	Unclear	Low	Low	Low	Low
Zheng et al, 2010	Unclear	Low	Unclear	Low	Unclear	Low	Low
Zhao et al, 2015	Unclear	Low	Unclear	Low	Low	Unclear	Low
Chi et al, 2012	Low	Unclear	Unclear	Low	Low	Low	Low
Guan et al, 2012	Low	Unclear	Unclear	Low	Low	Low	Low
Ming et al, 2014	Unclear	Low	Unclear	Low	Low	Low	Unclear
Xu et al, 2012	Low	Low	Unclear	Low	Low	Low	Unclear
Chen et al, 2011	Unclear	Low	Unclear	Low	Low	Unclear	Unclear
Zhang et al, 2013	Low	Low	Unclear	Low	Unclear	Low	Unclear
Zhang et al, 2011	Low	Unclear	Unclear	Low	Low	Low	Low
Li et al, 2009	Low	Low	Unclear	Low	Low	Low	Low
Guo et al, 2007	Unclear	Low	Unclear	Low	Low	Unclear	Low

Guo et al, 2008	Low	Low	Unclear	Low	Low	Low	Unclear
Guo et al, 2006	Low	Unclear	Unclear	Low	Unclear	Low	Unclear
Lv et al, 2012	Low	Low	Unclear	Low	Low	Low	Low
Wang et al, 2012	Low	Low	Unclear	Low	Low	Low	Low
Ming et al, 2014	Unclear	Unclear	Unclear	Low	Low	Low	Low
Zhao et al, 2010	Low	Unclear	Unclear	Low	Low	Low	Unclear
Zheng et al, 2013	Low	Low	Unclear	Low	Low	Low	Unclear
Deng et al, 2014	Unclear	Unclear	Unclear	Low	Low	Low	Low
Zhang et al, 2009	Low	Low	Unclear	Low	Low	Unclear	Low
Wu et al, 2013	Unclear	Unclear	Unclear	Low	Unclear	Low	Low
Lou et al, 2010	Low	Unclear	Unclear	Low	Low	Low	Low
Fu et al, 2010	Low	Low	Unclear	Low	Low	Low	Unclear
Gao et al, 2012	Low	Low	Unclear	Low	Low	Low	Low
Liu et al, 2013	Unclear	Unclear	Unclear	Low	Unclear	Low	Low
Bai et al, 2007	Low	Low	Unclear	Low	Unclear	Low	Unclear
Zhou et al, 2010	Unclear	Low	Unclear	Low	Low	Unclear	Low
Zhao et al, 2016	Unclear	Low	Unclear	Low	Low	Low	Low
Mou et al, 2013	Unclear	Low	Unclear	Low	Low	Low	Low
Kong et al, 2011	Unclear	Low	Unclear	Low	Low	Low	Low
Wang et al, 2008	Low	Low	Unclear	Low	Unclear	Low	Low
Zhong et al, 2009	Unclear	Low	Unclear	Low	Low	Low	Low
Wu et al, 2009	Low	Unclear	Unclear	Low	Low	Low	Unclear
Liu et al, 2010	Unclear	Low	Unclear	Low	Low	Low	Low
Chen et al, 2010	Unclear	Low	Unclear	Low	Low	Low	Low
Ge et al, 2014	Low	Low	Unclear	Low	Low	Low	Unclear
Wang et al, 2009	Unclear	Unclear	Unclear	Low	Unclear	Unclear	Low

Zhang et al, 2013	Low	Low	Unclear	Low	Low	Unclear	Unclear
Feng et al, 2011	Low	Low	Unclear	Low	Low	Low	Low
Liao et al, 2008	Low	Low	Unclear	Low	Low	Low	Low
Zhang et al, 2012	Low	Unclear	Unclear	Low	Low	Low	Low
Liu et al, 2014	Unclear	Low	Unclear	Low	Low	Unclear	Unclear
Cao et al, 2007	Low	Unclear	Unclear	Low	Unclear	Low	Low
Li et al, 2014	Low	Low	Unclear	Low	Low	Low	Low
Kim et al, 2008	Unclear	Low	Unclear	Low	Low	Low	Unclear
Zhang et al, 2016	Low	Unclear	Unclear	Low	Low	Unclear	Low
Zhang et al, 2016	Low	Low	Unclear	Low	Unclear	Low	Low
